# Protective Effects of Fisetin Against Oxidative Stress in Human Sperm: Implications for Cryopreservation

**DOI:** 10.3390/antiox15050583

**Published:** 2026-05-04

**Authors:** Sara Al-Mashharawi, Rahaf Dabe, Zina Al-Alami, Nadia Muhaidat, Mohammad H. Abukhalil, AbdelKader Battah, Mamoun Ahram

**Affiliations:** 1 Department of Medical Laboratory Sciences, Faculty of Allied Medical Sciences, Al-Ahliyya Amman University, P.O. Box 115, Amman 19111, Jordan; sarahmashhrawi@gmail.com; 2 Department of Biological Science, Faculty of Biology, The University of Jordan, Amman 11942, Jordan; rahafdabe@gmail.com; 3 Department of Obstetrics & Gynaecology, School of Medicine, The University of Jordan, Amman 11942, Jordan; n.mhaidat@ju.edu.jo; 4 Department of Biology, College of Science, Al-Hussein Bin Talal University, Ma’an 71111, Jordan; mabukhalil@ahu.edu.jo; 5 Department of Pathology Microbiology and Forensic Medicine, School of Medicine, The University of Jordan, Amman 11942, Jordan; akbattah@ju.edu.jo; 6 Department of Physiology and Biochemistry, School of Medicine, The University of Jordan, Amman 11942, Jordan; m.ahram@ju.edu.jo

**Keywords:** antioxidant, cryopreservation, fisetin, human sperm, hydrogen peroxide, sperm motility, sperm viability

## Abstract

**Background:** Cryopreservation induces the production of excessive reactive oxygen species (ROS), which decreases sperm physiological functions. Phytochemicals with antioxidant properties, such as fisetin, have shown promising results in reducing oxidative stress (OS). **Aim:** We aimed to evaluate whether fisetin can counteract the OS exerted on sperm. **Methodology:** Fisetin (15 and 30 µM) was tested on normozoospermic semen samples that were either frozen in liquid nitrogen or treated with H_2_O_2_ to induce OS. Sperm motility, sperm viability, mitochondrial membrane potential, metabolic activity, ROS content, lipid peroxidation, reduced glutathione, ATP contents, and apoptosis were tested and compared to controls. **Results:** The protective effect of fisetin on human sperm was observed against OS-induced stress. Fisetin significantly improved sperm motility, viability, mitochondrial and metabolic activity, and ATP content by reducing OS and lipid peroxidation. Fisetin reduced necrotic cell death and improved sperm survival under H_2_O_2_-OS. **Conclusions:** Fisetin protects human sperm from OS, with 30 µM showing greater effectiveness, supporting its potential use in sperm preservation and OS conditions. Further studies are needed to optimize its concentration, elucidate its mechanism of action, and confirm its putative use as an additive in sperm cryoprotective media.

## 1. Introduction

Sperm cryopreservation is a commonly practiced technique for preserving male fertility in humans and various animal species [1] During this process, the chemical and biological activities are slowed down [2]. Nevertheless, cryopreservation might adversely affect the physiology of sperm since it causes structural and physiological modifications that might lead to the eventual death of the sperm and poor quality after thawing, along with other variations in the physical properties of sperm among different species, such as sperm shape, size, and the lipid-protein ratio [3].

One of the most detrimental consequences of sperm cryopreservation is the excessive generation of reactive oxygen species (ROS) [4]. ROS include hydroxyl radical (HO^•^), superoxide anions (O_2_^•−^), nitric oxide (^•^NO), and lipid radicals. The other ROS that are not free radicals but still have oxidizing properties are hypochlorous acid (HOCl), hydrogen peroxide (H_2_O_2_), and peroxynitrite (ONOO¯) [5]. 

Under physiological conditions, ROS play a role in sperm acrosome reaction, capacitation, hyperactivation, and fertilization; however, excessive ROS production overwhelms endogenous antioxidant defenses, resulting in oxidative stress (OS) and cellular damage [6]. Nevertheless, sperm handling, washing, and cryopreservation may enhance or induce the level of OS in sperm samples, which may become a cause for concern, particularly if the levels of ROS and/or DNA damage in the basal semen sample are already increased [7]. 

Decreased sperm motility resulted from H_2_O_2_ treatment and freezing and thawing; the latter causes OS by producing a large amount of ROS, which affects sperm motility and induces autophagy, directing the sperm towards necrosis and apoptosis [8].

The major ROS found in human spermatozoa is H_2_O_2_. Moderately high concentrations of H_2_O_2_ immobilize sperm samples without affecting their viability, due to reduced phosphorylation of axonemal proteins and intracellular adenosine triphosphate (ATP) reduction. High concentrations of H_2_O_2_ stimulate lipid peroxidation and death [9].

Recent research has highlighted the therapeutic potential of plant-derived antioxidants, particularly phytochemicals such as polyphenols and flavonoids, which exhibit strong antioxidant properties [10]. Extracts rich in polyphenols and flavonoids have demonstrated significant capacity to neutralize OS and protect cellular integrity [11]. A recent meta-analysis further confirmed that antioxidant supplementation can reduce sperm DNA fragmentation, improve sperm parameters, and increase pregnancy rates [12]. Fisetin (3,3′,4′,7-tetrahydroxyflavone) is a naturally occurring flavonol found in fruits and vegetables such as apples, grapes, onions, tomatoes, and cucumbers [13]. Fisetin demonstrates anti-metastatic properties; it acts as an inhibitor of PI3K/Akt, enhances apoptosis, inhibits cell proliferation, relieves OS and inflammation by upregulating some enzymes like catalase, glutathione peroxidase, and superoxide dismutase, and increases anti-inflammatory cytokine [14].

Since H_2_O_2_ is commonly employed as an inducer of OS to evaluate the protective efficacy of various antioxidants on sperm parameters and biochemical indicators [15,16] and given the central role of OS in sperm dysfunction during cryopreservation and laboratory processing, fisetin emerges as a promising candidate for mitigating ROS-mediated damage. Therefore, the study aims to evaluate the protective effects of fisetin when used as an antioxidant additive in sperm cryoprotective media and to assess its efficacy in counteracting H_2_O_2_-induced OS and apoptosis in human sperm. The results could lead to the development of a better sperm cryopreservation method by exploring the potential of fisetin to enhance sperm quality, particularly by exploring the impact of fisetin on human sperm motility, viability, sperm activity, ROS content, and mitochondrial membrane potential after the freezing–thawing cycle and after incubation with H_2_O_2_ as an OS inducer condition.

## 2. Materials and Methods

### 2.1. Ethical Approval

This study was performed according to ethical standards and was approved by the Institutional Review Board (IRB) of the Jordan University Hospital (JUH) (approval numbers 10/2024/13274 provided on 21 May 2024 and 10/2024/14472 provided on 11 June 2024). All participants gave written informed consent before their inclusion in the study.

### 2.2. Sample Collection

A total of 108 semen samples were collected from the routine lab and the Assisted Reproductive Unit of the Jordan University Hospital during the year 2024. Of these, 54 samples were allocated to the H_2_O_2_ experiment while the remaining 54 samples were assigned to the cryopreservation experiment. All the study participants included in the study had normozoospermic semen and had no chronic diseases and did not consume medicines and/or supplements on a daily basis. Participants with abnormal semen samples, those who have chronic diseases, or those taking any regular medication were excluded. The age range was 20 to 50 years old.

### 2.3. Sample Preparation

Semen samples were collected by masturbation following three to five days of abstinence from sexual activity. All semen analyses, including the assessment of sperm motility and viability, were performed according to the 5th edition of the World Health Organization (WHO) criteria [17]. After liquefaction, semen samples were analyzed for volume, sperm concentration, motility, morphology, and total sperm count, with sperm motility further categorized into progressive, non-progressive, and immotile patterns based on movement characteristics. Only samples meeting WHO criteria for normospermia were included in the study. Subsequently, high-quality motile sperm were isolated using the swim-up technique for experimental procedures to ensure consistency and reliability of the experimental outcomes.

Briefly, liquefied semen samples were transferred into sterile conical centrifuge tubes and centrifuged at 1750 rpm for 10 min. The supernatant was carefully discarded without disturbing the sperm pellet. Subsequently, 0.5–1.0 mL of sperm washing medium was gently layered along the inner wall of the tube to form a distinct upper phase above the pellet. The tubes were then inclined at approximately 45° and incubated at 37 °C for 45–60 min without disturbance. During this incubation period, highly motile and morphologically normal spermatozoa actively migrated from the pellet into the upper layer of the medium. After incubation, the upper fraction (approximately 0.3–0.5 mL), enriched with motile sperm, was carefully aspirated using a sterile pipette and transferred into a new sterile tube. The recovered sperm fraction was then re-evaluated for concentration, motility, and morphology prior to use in experimental procedures.

Sperm samples were processed individually per donor and were not pooled. Each experimental tube contained a defined total sperm count that was adjusted by dilution with sperm wash medium to meet the requirements of each specific assay, with counts ranging from 2 × 10^5^ to 1 × 10^6^ sperm per tube, according to the requirements of the measured test. For each individual experiment, all treatment groups were standardized to the same sperm concentration to ensure valid comparisons.

### 2.4. Fisetin Preparation

A stock solution of 5 mM fisetin (2-(3,4-Dihydroxyphenyl)-3,7-dihydroxy-4H-chromen-4-one hydrate; purity 98%; GenoChem World, Valencia, Spain) was prepared by dissolving 1.5 mg of fisetin in 1 mL dimethyl sulfoxide (DMSO; cell culture grade; CooperSurgical, Trumbull, CT, USA), in accordance with its hydrophobic nature. The stock solution was freshly prepared and serially diluted in sperm washing medium to obtain a range of working concentrations (1250, 625, 312.5, 156.25, 78.125, 39.1, 19.53, 9.76, 4.88, 2.44 μM). Care was taken to ensure that the final concentration of DMSO in all treatments remained below 1.0% to avoid solvent-related effects on sperm function. To determine the non-toxic concentration range of fisetin, a dose–response assessment was conducted using sperm samples obtained after the swim-up selection of motile spermatozoa. Briefly, 200 μL of sperm suspension (adjusted to approximately 1 × 10^6^ spermatozoa per sample) was incubated with 50 μL of each fisetin dilution for 1 h at 37 °C under controlled conditions. The final concentrations of Fisetin were 250, 125, 62.5, 31.25, 15.6, 7.8, 3.9, 1.9, 0.98, and 0.48 μM. Untreated samples containing sperm washing medium alone were used as negative controls.

Following incubation, sperm motility and viability were evaluated using standard laboratory procedures according to the World Health Organization. The assessment was performed on at least two independent samples to confirm reproducibility. The selection of fisetin concentrations also ensured a sufficient safety margin while minimizing any potential confounding effects associated with DMSO exposure [18,19].

### 2.5. Study Design

**Figure 1:**
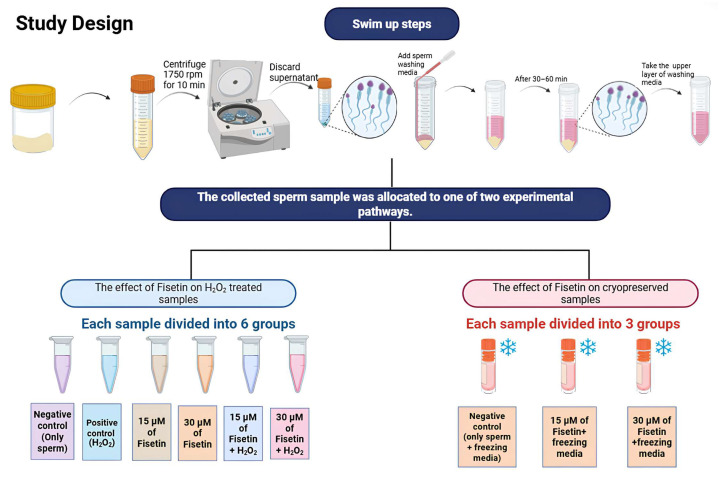
Graphical representation for the study design (created using Biorender.com), the arrows represent the successive steps.

This study was divided into two parts (Figure 1). The first part was designed to investigate the effect of H_2_O_2_-induced OS on sperm, as well as the potential protective effect of fisetin. The second part was designed to investigate the effects of cryopreservation in liquid nitrogen and subsequent thawing on sperm, as well as the effect of fisetin.

#### 2.5.1. Preparation of H_2_O_2_ and Experimental Groups to Assess the Effect of Fisetin on H_2_O_2_-Induced OS on Sperm

A final H_2_O_2_ concentration of 100 µmol/L was chosen based on previous research that showed its ability to promote OS in sperm [20]. Semen samples were obtained from 54 healthy donors. For each experimental endpoint, a subset of six independent donors was used. Each semen sample was divided into six equal aliquots and subjected to the following experimental conditions using high-quality sperm selected by the swim-up technique. Group 1, negative control (−ve) selected sperm in 200 μL of sperm wash medium and 60 μL of additional sperm wash medium. Group 2, positive control (+ve), selected sperm in 200 μL of sperm wash medium mixed with 10 μL of H_2_O_2_ (final concentration 100 μM) and 50 μL of sperm wash medium. Group 3, a low dose of fisetin (15 μM), mixed 200 μL of sperm wash media with 50 μL of fisetin (final concentration 15 μM) and 10 μL of sperm wash medium. Group 4, a high dose of fisetin (30 μM), mixed 200 μL of sperm wash medium, 50 μL fisetin (final concentration 30 μM), and 10 μL of sperm wash medium. Group 5, a low dose of fisetin with H_2_O_2_ (15 μM—H_2_O_2_), mixed 200 μL of sperm wash media, 50 μL fisetin (final concentration 15 μM), and 10 μL of H_2_O_2_ (final concentration 100 μM). Group 6, a high dose of fisetin with H_2_O_2_ (30 μM—H_2_O_2_), mixed 200 μL of sperm wash media, 50 μL fisetin (final concentration 30 μM), and 10 μL of H_2_O_2_ (final concentration 100 µmol/L).

All groups were incubated at 37 °C for one hour. Following incubation, cells were washed with phosphate-buffered saline (PBS), the supernatant was discarded, and the samples were prepared for subsequent experimental analyses.

#### 2.5.2. Sperm Cryopreservation and Thawing and Experimental Groups to Assess the Effect of Fisetin on Sperm Cryopreservation

Semen samples were obtained from 54 healthy donors. For each experimental end-point, a subset of six independent donors was used. Each semen sample was divided into three equal aliquots and subjected to the following experimental conditions using high-quality sperm selected by the swim-up technique. Group 1, the negative control, was not treated. In Group 2, the sperm sample was treated with a low concentration of fisetin (15 µM). In Group 3, the sperm sample was treated with a high concentration of fisetin (30 µM).

To prepare the samples for cryopreservation, the high-quality sperm were frozen within sperm freezing media (Quinn’s Advantage ™ Sperm Freezing Medium, CooperSurgical Company, Trumbull, USA), and the freezing process was performed following the kit instructions. In summary, the treated semen samples were mixed with an equal volume of sperm freezing media (1:1), supplemented with two concentrations of fisetin (to reach a final concentration of 30 µM and 15 µM, respectively), and sperm freezing media only (as a negative control). According to the kit, each sample mixture was placed 3 cm above the liquid nitrogen level for 30 min until it became frozen in the nitrogen vapor. Then all samples were transferred into liquid nitrogen to be stored at −196 °C.

Sperm was tested after two weeks to four weeks of freezing; the duration was determined according to Alqawasmeh (2021) [21]. The thawing process was performed by removing the cryovials from liquid nitrogen and placing them into a 37 °C water bath for 10 min according to the manufacturer’s instructions.

### 2.6. Effects of Fisetin on Sperm Parameters, OS Biomarkers, Energy Metabolism, and Mitochondrial Function

The following experiments were done on the previously mentioned groups to assess the effect of fisetin on sperm cryopreservation and induced OS.

#### 2.6.1. Sperm Motility

The percentage of motile sperm was determined manually using a microscope, and sperm motility was classified into three groups: progressive, non-progressive, and immotile. Motility was recorded both after the swim-up procedure and post-treatment for further analysis and comparison.

#### 2.6.2. Sperm Viability

The percentage of sperm viability was calculated by categorizing the sperm as alive or dead according to their uptake of the dye using the SpermVit kit (Antigenes GmbH, Schwelm, Germany).

#### 2.6.3. Reactive Oxygen Species (ROS) Levels

The ROS levels present in sperm were measured by using a ROS assay kit (GenoChem World, Valencia, Spain). This assay is based on a test of the fluorescent probe 2′,7′-dichlorofluorescin diacetate (DCFH-DA) which is going to get deacetylated by esterases, once inside the cell, to form DCFH. This DCFH is oxidized by ROS to become fluorescent 2′,7′-dichlorofluorescein (DCF). Approximately, 1 × 10^6^ sperm per sample was used for the assay. Fluorescence was measured using a microplate reader (Synergy HTX multimode microplate reader (BioTek Instruments, Winooski, VT, USA)) at 488 nm excitation and 525 nm emission to quantify the ROS levels present in the cells.

#### 2.6.4. Lipid Peroxidation

To evaluate the impact of fisetin on lipid peroxidation, the malondialdehyde (MDA) content of the sperm samples was determined. Approximately, 1 × 10^6^ sperm per sample were used for the assay. A microplate reader (Synergy HTX multimode microplate reader (BioTek Instruments, Winooski, VT, USA)) was utilized to measure the absorbance of the solution at 534 nm; this reading is an indicator of the damage that resulted from the OS on the sperm cell membrane.

#### 2.6.5. Reduced Glutathione (GSH) Levels

A reduced glutathione (GSH) content assay kit (GenoChem World, Valencia, Spain) was used to detect the effect of fisetin on GSH content in the treated groups with and without stress. Approximately, 1 × 10^6^ sperm per sample were used for the assay. The absorbance was measured by a microplate reader (Synergy HTX multimode microplate reader (BioTek Instruments, Winooski, VT, USA)) at 412 nm, and the net change in absorbance was calculated.

#### 2.6.6. Analysis of Sperm Metabolic Activity

The resazurin (7-hydroxy-3H-phenoxazin-3-one10-oxide) salt test was used to analyze the sperm metabolic activity. Approximately, 1 × 10^6^ sperm per sample was used for the assay. The fluorescent compound resorufin results after reducing resazurin by metabolically active sperm cells; this test results in a reliable indicator of sperm metabolic activity. The fluorescent resorufin was measured by a multimode microplate reader (Synergy HTX multimode microplate reader (BioTek Instruments, Winooski, VT, USA)) at a 560 nm excitation wavelength and 590 nm emission wavelength.

#### 2.6.7. Analysis of JC-1 Assay for Mitochondrial Membrane Potential

To measure the mitochondrial membrane potential in sperm cells, JC-1 dye (5,5,6,6-Tetrachloro-1,1,3,3-tetraethyl-imidacarbocyanine iodide) (Biosynth Ltd., Newbury, UK) was used. Approximately, 1 × 10^6^ sperm per sample was used for the assay. This compound emits fluorescence in the red and green spectra. Red fluorescence represents the J-aggregate form of the compound to indicate a high mitochondrial membrane potential. On the contrary, green fluorescence represents the monomer form of the compound and indicates a low mitochondrial membrane potential. The higher the red to green fluorescence intensity ratios, the higher the membrane potential and the healthier the sperm mitochondria.

The fluorescence for the monomer was measured at a 485 nm excitation wavelength and 528 nm emission wavelength while the J-aggregate was measured at a 485 nm excitation wavelength and 590 nm emission wavelength by a microplate reader (Synergy HTX multimode microplate reader (BioTek Instruments, Winooski, VT, USA)). The ratio of the J-aggregate/monomer that represents the hyperpolarization/depolarization state of the mitochondria was calculated.

#### 2.6.8. ATP Content Assay Kit

For the evaluation of the ATP content in the sperm samples, a commercially available ATP Activity Assay kit was used (GenoChem World, Valencia, Spain). Approximately 1 × 10^6^ sperm per sample were used for the assay. The used assay is based on measuring NADPH absorbance at 340 nm; this value is proportional to the ATP content. When the absorption reaction of NADPH is low, the ATP level is also low. After following the manufacturer’s instructions, a standard curve was prepared, and the absorbance of each tube was recorded by a Synergy HTX multimode microplate reader (BioTek Instruments, Winooski, VT, USA) at 340 nm.

#### 2.6.9. Apoptosis Analysis

The apoptosis process was detected using the TACS^TM^ Annexin V-FITC Kit USA R&D systems Inc., Minneapolis, MN, USA)) and processed by a flow cytometer. Approximately, 2 × 10^5^ sperm per sample were used for the assay.

### 2.7. Statistical Analysis

**Figure 2:**
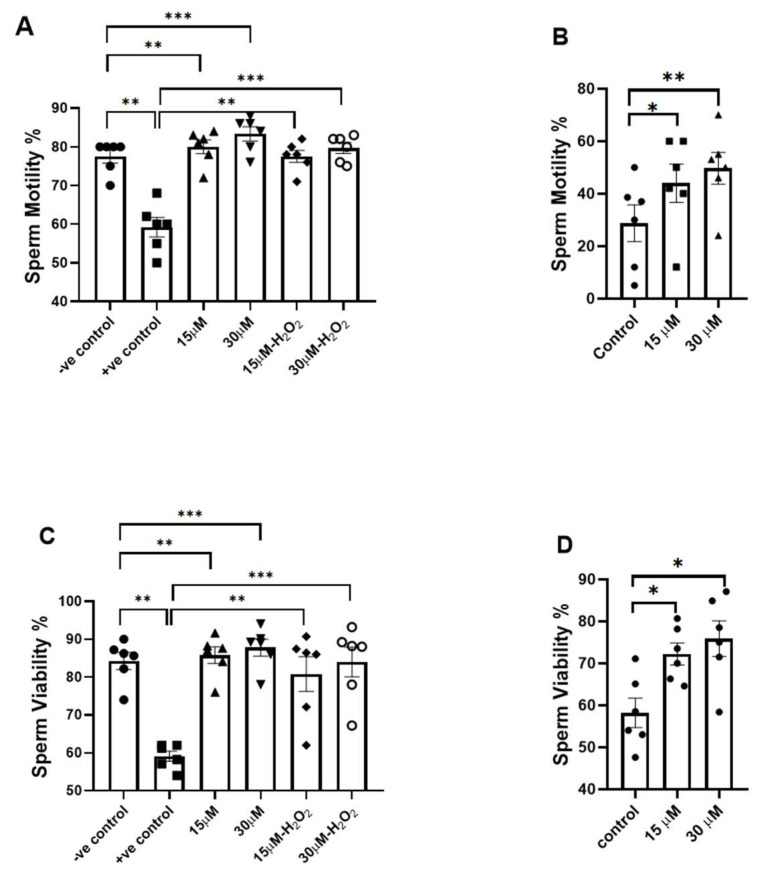
Effects of fisetin on sperm motility (**A**,**B**) and viability (**C**,**D**). (**A**,**C**) represent control (−H_2_O_2_) and oxidative stress (+H_2_O_2_) conditions. (**B**,**D**) represent post freeze–thaw conditions. For each concentration, the *p*-value was determined by comparing the group with and without treatment. Data are presented as mean ± SEM; * *p* < 0.05, ** *p* < 0.01, and *** *p* < 0.001.

**Figure 3:**
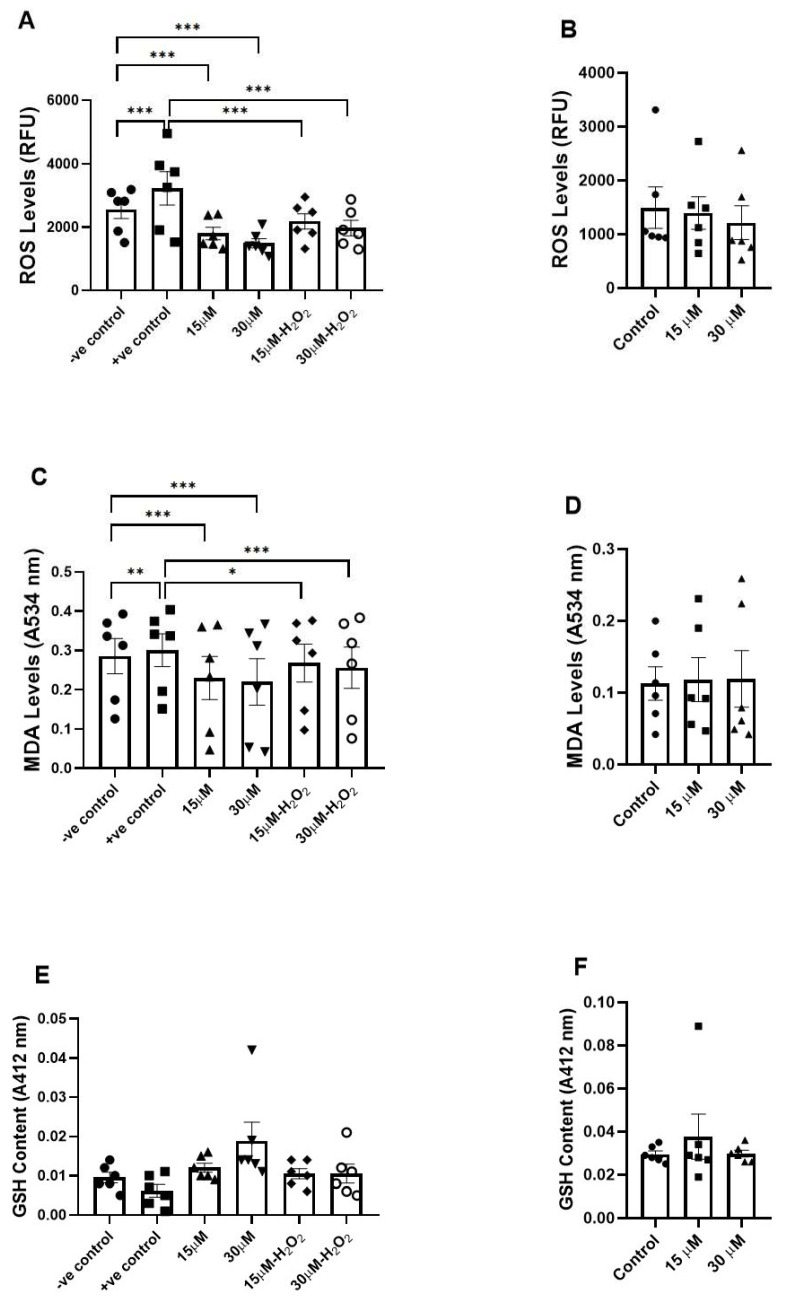
Effects of fisetin on oxidative stress biomarkers. (**A**,**C**,**E**) show ROS levels ((**A**); RFU), lipid peroxidation ((**C**); MDA at 534 nm), and GSH content (**E**) under control (−H_2_O_2_) and oxidative stress (+H_2_O_2_) conditions, with and without fisetin treatment (15 and 30 µM). (**B**,**D**,**F**) show ROS levels (**B**), lipid peroxidation ((**D**); MDA), and GSH content (**F**) after freeze-thawing, with and without fisetin treatment. For each concentration, the *p*-value was determined by comparing the group with and without treatment. Data are presented as mean ± SEM; * *p* < 0.05, ** *p* < 0.01, and *** *p* < 0.001. RFU: relative fluorescence units.

**Figure 4:**
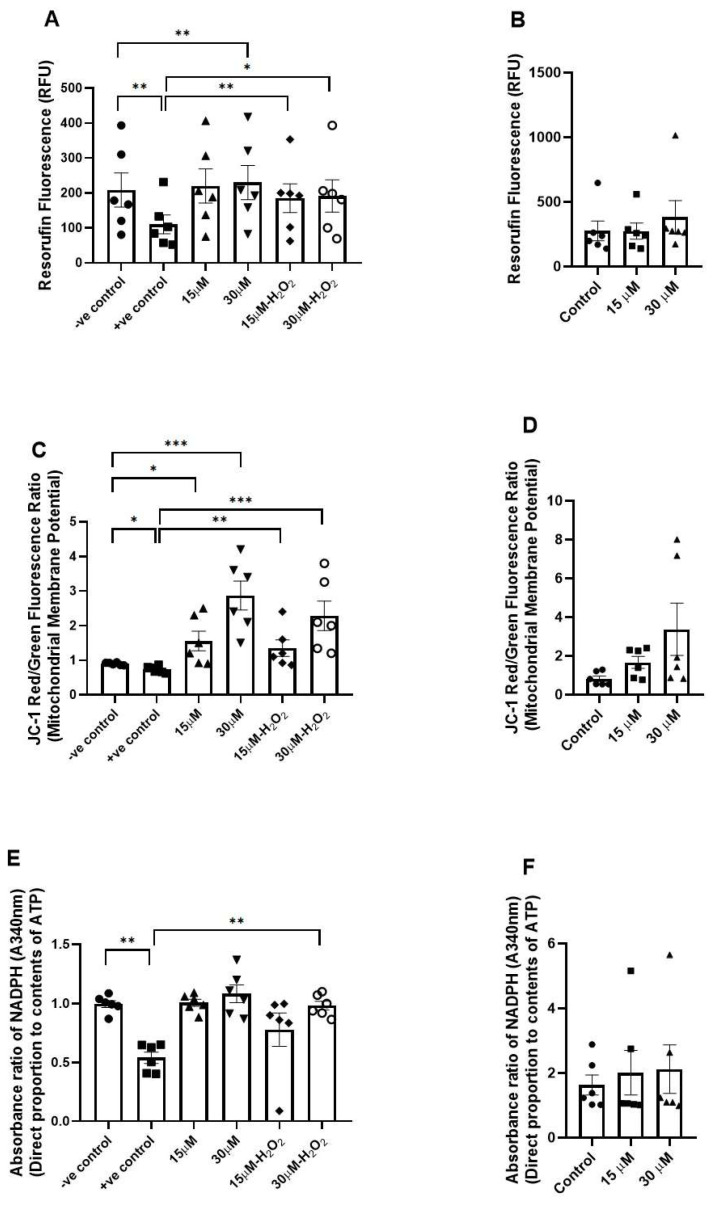
Assessment of energy metabolism and mitochondrial function. (**A**) shows metabolic activity of sperm measured by the resazurin reduction assay and quantified as resorufin fluorescence (Ex 560 nm/Em 590 nm) under control (−H_2_O_2_) and oxidative stress (+H_2_O_2_) conditions with and without fisetin treatment (15 and 30 µM), and (**B**) shows after freeze–thawing. (**C**) shows mitochondrial membrane potential assessed by JC-1 red/green fluorescence ratio under control and oxidative stress conditions with and without fisetin, and (**D**) shows after freeze–thawing. (**E**) shows NADPH absorbance (A340 nm), reflecting ATP-related metabolic activity, under control and oxidative stress conditions with and without fisetin treatment, and (**F**) shows after freeze–thawing. For each concentration, the *p*-value was determined by comparing the group with and without treatment. Data are presented as mean ± SEM; * *p* < 0.05, ** *p* < 0.01, and *** *p* < 0.001. RFU: relative fluorescence units.

**Figure 5:**
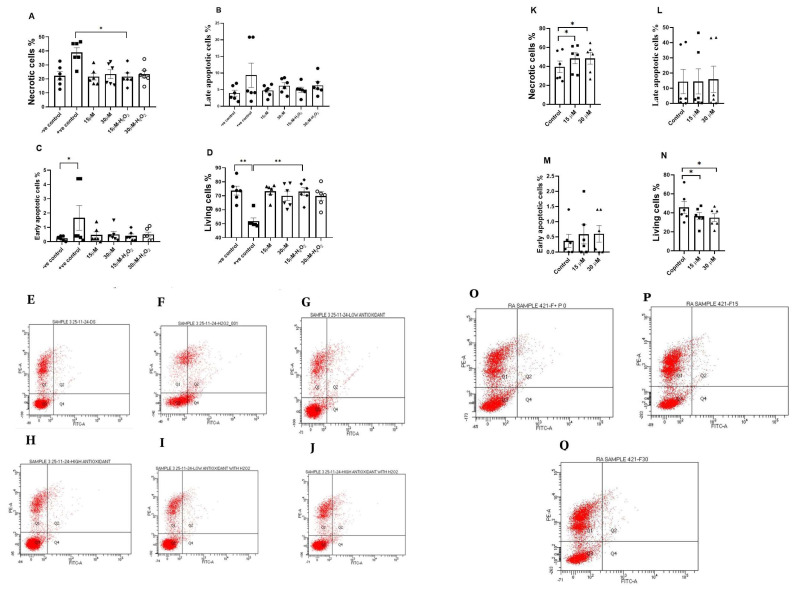
Effects of fisetin on apoptosis assessed using the TACS Annexin V apoptosis detection kit. Cell death was evaluated based on Annexin V-FITC/propidium iodide (PI) staining, and samples were categorized into necrotic, late apoptotic, early apoptotic, and living cells. (**A**–**D**) represent control (−H_2_O_2_), oxidative stress (+H_2_O_2_), and fisetin treatment (15 and 30 µM) conditions. (**K**–**N**) represent post freeze–thaw conditions with and without fisetin treatment. For each concentration, the *p*-value was determined by comparing the group with and without treatment. Data are presented as mean ± SEM; * *p* < 0.05 and ** *p* < 0.01. (**E**–**J**,**O**–**Q**) show the representative flow cytometry dot plots for: (**E**) control (−H_2_O_2_), (**F**) +H_2_O_2_, (**G**) fisetin 15 µM, (**H**) fisetin 30 µM, (**I**) fisetin 15 µM + H_2_O_2_, (**J**) fisetin 30 µM + H_2_O_2_, (**O**) post-freeze–thaw control, (**P**) fisetin 15 µM post freeze–thaw, and (**Q**) fisetin 30 µM post freeze–thaw.

Results were expressed as mean ± standard error of the mean (SEM). Statistical analyses were performed using R software (version 4.4.3; R Foundation for Statistical Computing, Vienna, Austria). Data were analyzed using the Friedman test followed by Conover’s test for pairwise comparisons with Holm-adjusted *p*-values. Graphical representations were prepared using GraphPad Prism (version 8; GraphPad Software, San Diego, CA, USA). A *p* < 0.05 was considered statistically significant. Asterisks (*) are used in Figure 2, Figure 3, Figure 4 and Figure 5 to indicate statistical significance where * means *p* < 0.05, ** means *p* < 0.01, and *** means *p* < 0.001.

## 3. Results

### 3.1. Assessment of the Dose-Dependent Effect of Fisetin on Sperm Function

The results demonstrated a clear concentration-dependent effect of fisetin on sperm function. Higher concentrations (≥125 μM) impaired sperm motility and viability to approximately 20–50% of control values. Intermediate concentrations (31.25–62.5 μM) exhibited moderate reductions, while lower concentrations (≤15.6 μM) maintained sperm motility and viability at levels comparable to controls. Based on these findings, 30 μM was identified as the highest concentration that did not induce significant adverse effects on sperm motility, viability, or morphology. Therefore, concentrations of 15 μM and 30 μM were selected as the low and high non-toxic working doses, respectively, for subsequent experiments.

### 3.2. Effects of Fisetin on Sperm Motility and Viability

The induction of OS with H_2_O_2_ significantly reduced sperm motility (*p* < 0.01), treatment with fisetin at 15 µM and 30 µM significantly increased sperm motility both without OS (*p* < 0.01 and *p* < 0.001, respectively) and under H_2_O_2_-induced OS (*p* < 0.01 and *p* < 0.001, respectively; Figure 2A). In addition, after freezing and thawing, fisetin (15 µM and 30 µM) significantly increased the percentage of motility (*p* < 0.05 and *p* < 0.01, respectively; Figure 2B).

Induction of OS by H_2_O_2_ significantly reduced sperm viability (*p* < 0.01). Treatment with fisetin at 15 µM and 30 µM significantly improved sperm viability in the absence of OS (*p* < 0.01 and *p* < 0.001, respectively) and in the presence of OS (*p* < 0.01 and *p* < 0.001, respectively; Figure 2C). In addition, after freezing and thawing, both concentrations increased the percentage of sperm viability (*p* < 0.05; Figure 2D).

### 3.3. Assessment of the Effects of Fisetin on Oxidative Stress Biomarkers

H_2_O_2_ exposure significantly increased sperm ROS levels (*p* < 0.001), yet both fisetin concentrations (15 µM and 30 µM) significantly reduced sperm ROS levels, both without OS and under H_2_O_2_-induced OS (*p* < 0.001; Figure 3A). Moreover, H_2_O_2_ exposure significantly increased lipid peroxidation, as indicated by elevated MDA levels (*p* < 0.01). Fisetin significantly attenuated lipid peroxidation both in the absence (15 µM and 30 µM; *p* < 0.001) and presence of OS (*p* < 0.05 and *p* < 0.001, respectively; Figure 3C). As for GSH levels, no significant differences were observed among the groups, both in the absence and presence of H_2_O_2_-induced OS (Figure 3E). Furthermore, no significant changes were observed in sperm ROS, MDA, and GSH levels following freezing and thawing in fisetin-treated samples compared with the control (Figure 3B,D,F).

### 3.4. Assessment of the Effects of Fisetin on Energy Metabolism and Mitochondrial Function

Induction of OS using H_2_O_2_ resulted in a significant reduction in sperm metabolic activity, as indicated by decreased resorufin fluorescence (*p* < 0.01). Fisetin (30 µM) increased sperm metabolic activity without OS (*p* < 0.01), but both fisetin concentrations (15 µM with *p* < 0.01 and 30 µM with *p* < 0.05) increased the metabolic activity after OS (Figure 4A).

H_2_O_2_ significantly (*p* < 0.05) reduced the mitochondrial membrane potential, yet fisetin could significantly attenuate that reduction (15 µM with *p* < 0.05 and 30 µM with *p* < 0.001; Figure 4C).

The ATP content in sperm was significantly (*p* < 0.01) reduced after H_2_O_2_ treatment, indicating that OS negatively affects cellular energy metabolism; nevertheless, fisetin (30 µM) could overcome this reduction (*p* < 0.01; Figure 4E).

No significant changes were observed in sperm metabolic activity, mitochondrial membrane potential, or ATP content following freezing and thawing in fisetin-treated samples compared with the control (Figure 4B,D,F).

### 3.5. Apoptosis Detection Test

Exposure of sperm to H_2_O_2_ significantly increased the percentage of early apoptotic sperm (*p* < 0.05; Figure 5C), showed a non-significant increase in necrotic cells, and significantly reduced the proportion of living sperm (*p* < 0.01; Figure 5D). In fisetin treatment under H_2_O_2_- induced OS, 15 µM fisetin significantly reduced necrotic sperm (*p* < 0.05) and increased the percentage of living cells (*p* < 0.01; Figure 5A–D).

Following freeze-thawing, fisetin treatment was associated with changes in sperm cell distribution. Both 15 µM and 30 µM concentrations showed an increase in the percentage of necrotic cells compared with the control (*p* < 0.05; Figure 5K). In parallel, the proportion of living sperm was reduced in fisetin-treated groups compared with the control after freeze-thawing (*p* < 0.05; Figure 5N).

In the dot plot, the X-axis represents the log of the Annexin V-FITC fluorescence, whereas the *Y*-axis displays the propidium iodide fluorescence (Figure 5E–J,O–Q). Early apoptotic cells are shown as a population of cells with bound Annexin V (right lower quadrant). Whereas late apoptotic or necrotic cells are Annexin V-positive cells which take up propidium iodide (right upper quadrant). In addition, normal viable cells’ population that are negative for both propidium iodide and Annexin V might also be present (left lower quadrant).

## 4. Discussion

The OS is a crucial factor impairing male fertility, arising when the balance between ROS production and antioxidant defenses is disrupted. Excessive ROS negatively affects sperm viability, motility, concentration, and functional competence, primarily through lipid peroxidation and damage to cellular membranes, proteins, and DNA [22,23].

Both experimental models used in this study—H_2_O_2_ exposure and cryopreservation—are well-established inducers of OS in spermatozoa. H_2_O_2_ exposure directly in-creased ROS and MDA levels, while significantly reducing sperm motility, viability, mitochondrial membrane potential, ATP production, and metabolic activity. These findings are consistent with previous reports indicating that sperm membranes, due to their high polyunsaturated fatty acid content, are particularly susceptible to ROS-induced lipid peroxidation, leading to functional impairment [24]. Similar oxidative damage has been documented in both human and buffalo sperm subjected to OS, resulting in structural and functional deterioration [25,26].

Current results revealed that fisetin has protective effects on human sperm under oxidative and cryopreservation stress. The treatment with H_2_O_2_ resulted in a significant reduction in sperm motility, viability, mitochondrial activity, metabolic activity, ATP content, and increased ROS levels, lipid peroxidation, and early apoptosis. In contrast, treatment with fisetin showed a significant improvement in sperm motility, viability, metabolic activity, mitochondrial membrane potential, and ATP content. This was due to the reduction in ROS levels and lipid peroxidation. The GSH levels did not show any changes. However, fisetin appeared to effectively reduce oxidative damage, reduced necrotic cell death, and increased viable sperm under OS. Therefore, fisetin has a promising role in protecting human sperm, by having significant antioxidant properties.

Previous studies showed that many flavonoids served as promising additives to ex-tenders, to reduce oxidative stress and improve the efficiency of storage and cryopreservation, such as quercetin [27], kaempferol [28], apigenin [29], luteolin [30], hesperidin [31], naringenin [32], and epigallocatechin-3-gallate [33].

The present study showed that cryopreservation-induced OS significantly impairs sperm quality, confirming a previously reported scientific finding regarding particularly the impairment of motility and viability [4]. Consistent with earlier studies reporting post-thaw reductions in total and progressive motility and sperm parameters, the current results demonstrated a reduction in total motility upon freezing and thawing [34,35,36,37].

Fisetin, a bioactive flavonoid with potent antioxidant properties, demonstrated protective effects against OS in both experimental conditions. Fisetin is known to exert its antioxidant activity through non-enzymatic free radical scavenging, metal ion chelation, and participation in oxidoreductase reactions, thereby limiting oxidative damage to lipids, proteins, and DNA. Its antioxidant mechanism is primarily attributed to its hydroxyl groups, particularly the 3′ and 4′-OH groups in the B-ring, which donate electrons to neutralize free radicals [38,39].

Fisetin significantly reduced ROS accumulation and preserved cellular viability under OS, consistent with previous articles [40,41]. In the H_2_O_2_-induced OS model, both 15 µM and 30 µM concentrations improved sperm motility and viability and reduced ROS and MDA levels, mitochondrial membrane potential, and metabolic activity, while only the higher concentration significantly enhanced ATP production. These results are consistent with prior studies demonstrating that fisetin improved mitochondrial function under OS [38]. Similar protective effects of fisetin have been reported in models of reproductive toxicity induced by monosodium glutamate and long-term scrotal hyperthermia, further supporting its role in safeguarding spermatogenesis and sperm function [42,43].

Mitochondrial dysfunction is a key mechanism underlying OS-induced sperm impairment. Exposure to H_2_O_2_ significantly reduced mitochondrial membrane potential and ATP production, which are essential for flagellar movement and sperm motility. The present data support a previous report that concluded that H_2_O_2_ increases intracellular calcium levels, triggering mitochondrial permeability transition pore opening, mitochondrial membrane potential dissipation, and activation of apoptotic pathways [44]. Currently, OS is accompanied by reduced ATP levels, impaired motility, and increased oxidative damage. Fisetin supplementation counteracted these effects by stabilizing mitochondrial function and restoring energy production, thereby enhancing sperm motility.

Interestingly, while fisetin protected sperm function under OS, it appeared to modulate apoptosis-related pathways, consistent with reports describing its regulatory effects on apoptosis-related factors in other cellular systems [14,45].

Finally, fisetin enhanced sperm metabolic activity, as demonstrated by resazurin reduction assays. This observation aligns with previous reports indicating that fisetin improves metabolic health by modulating inflammation, energy metabolism, and cell survival pathways [46].

However, despite these valuable findings, there are certain limitations that should be considered, such as the use of short-term effects of fisetin without evaluating its effects on longer storage periods, and that molecular pathway analyses were not included, which limits the mechanistic interpretation. Future studies involving other fisetin concentrations should be carried out. Future investigations should also involve clinical trials to determine whether the addition of fisetin to cryopreservation media could be applicable and valid.

## 5. Conclusions

Fisetin has shown protective effects on human sperm against OS. It has shown improvement in essential functional parameters, which include motility, viability, metabolic, and mitochondrial function, by scavenging free radicals and reducing lipid peroxidation, but without altering the GSH levels. The current study has shown the promising use of fisetin, especially at higher concentrations, as an antioxidant supplement to improve the quality of sperm in cryopreservation and OS conditions.

## Data Availability

The original contributions presented in this study are included in the article.
